# CTP synthase polymerization in germline cells of the developing *Drosophila* egg supports egg production

**DOI:** 10.1242/bio.050328

**Published:** 2020-07-21

**Authors:** Jacqueline C. Simonet, Maya J. Foster, Eric M. Lynch, Justin M. Kollman, Emmanuelle Nicholas, Alana M. O'Reilly, Jeffrey R. Peterson

**Affiliations:** 1 Cancer Biology Program, Fox Chase Cancer Center, 333 Cottman Avenue, Philadelphia, PA 19111, USA; 2 Immersion Science Program, Fox Chase Cancer Center, 333 Cottman Avenue, Philadelphia, PA 19111, USA; 3 Department of Biochemistry, University of Washington, Seattle, WA 98195, USA; 4 Molecular Therapeutics Program, Fox Chase Cancer Center, 333 Cottman Avenue, Philadelphia, PA 19111, USA

**Keywords:** Metabolic enzyme, Nucleotide biosynthesis, Agglomeration

## Abstract

Polymerization of metabolic enzymes into micron-scale assemblies is an emerging mechanism for regulating their activity. CTP synthase (CTPS) is an essential enzyme in the biosynthesis of the nucleotide CTP and undergoes regulated and reversible assembly into large filamentous structures in organisms from bacteria to humans. The purpose of these assemblies is unclear. A major challenge to addressing this question has been the inability to abolish assembly without eliminating CTPS protein. Here we demonstrate that a recently reported point mutant in CTPS, Histidine 355A (H355A), prevents CTPS filament assembly *in vivo* and dominantly inhibits the assembly of endogenous wild-type CTPS in the *Drosophila* ovary. Expressing this mutant in ovarian germline cells, we show that disruption of CTPS assembly in early stage egg chambers reduces egg production. This effect is exacerbated in flies fed the glutamine antagonist 6-diazo-5-oxo-L-norleucine, which inhibits *de novo* CTP synthesis. These findings introduce a general approach to blocking the assembly of polymerizing enzymes without eliminating their catalytic activity and demonstrate a role for CTPS assembly in supporting egg production, particularly under conditions of limited glutamine metabolism.

This article has an associated First Person interview with the first author of the paper.

## Abstract (precis)

**Summary:** A point mutation in the nucleotide biosynthetic enzyme CTP synthase dominantly inhibits its polymerization and can be used to suppress CTPS assembly in a tissue-specific manner *in vivo*. Disrupting assembly in ovarian germline cells reduces egg production, demonstrating a functional role for CTPS assembly.

## INTRODUCTION

CTP synthase (CTPS) is an essential enzyme mediating *de novo* pyrimidine synthesis by catalyzing the ATP-dependent amination of UTP into CTP. As expected for a rate-limiting enzyme in a critical biosynthetic pathway, CTPS is subject to numerous types of post-transcriptional regulation including allosteric regulation by nucleotides (e.g. GTP) (Habrian et al., 2016; Lunn et al., 2008), post-translational modification (phosphorylation) (Chang et al., 2007; Kassel et al., 2010) and assembly into homomeric micron-scale polymers (Barry et al., 2014; Ingerson-Mahar et al., 2010; Liu, 2010; Noree et al., 2010). The role of polymerization has been particularly enigmatic (Liu, 2011), though it is reported to enhance the stability of the CTPS protein (Sun and Liu, 2019). Bacterial and eukaryotic CTPS exists basally in a tetrameric form, which can further assemble into linear protofilaments (Barry et al., 2014; Lynch et al., 2017). Intriguingly, bacterial and eukaryotic CTPS assemble into structurally distinct protofilaments with bacterial CTPS assemblies exhibiting reduced catalytic activity (Barry et al., 2014) while assemblies of human CTPS1 exhibit greater activity than their basal tetramers (Lynch et al., 2017). Adding to the complexity, within cells of both kingdoms, polymers of CTPS tetramers (protofilaments) can associate laterally into much larger filamentous macromolecular bundles, which can be tens of microns in length and hundreds of nanometers in width (Liu, 2011). Furthermore, other proteins not directly involved in pyrimidine biosynthesis have been identified that can co-assemble with CTPS in these larger structures *in vivo* (Chang et al., 2018; Keppeke et al., 2015; Strochlic et al., 2014).

Whether assembly into these ultrastructures is important for CTPS function is unknown, in part because of a lack of tools to disrupt the assembly of CTP filaments in their native context completely but without eliminating CTPS catalytic activity. A possible tool that could address this is a CTPS point mutant that prevents CTPS assembly and several such mutants have been previously reported, including A20R (Huang et al., 2017), R294D (Lin et al., 2018) and Histidine 355A (Lynch et al., 2017). However, a major challenge in interpreting experiments with these mutants is that, with the exception of R294D (Lin et al., 2018), it is not known whether expression of these non-assembling mutants also affects the assembly of endogenous, wild-type CTPS. As a result, cells could express a mixture of diffuse mutant CTPS and polymerized wild-type CTPS.

To develop a tool to dominantly inhibit endogenous CTPS assembly in live organisms, we focused on characterizing the effects of the H355A mutation. H355A is located in the CTPS GAT domain helical insert, which mediates CTPS polymerization (Lynch et al., 2017). Recombinant human CTPS1^H355A^ assembles into structurally normal tetramers but these tetramers cannot further assemble into linear protofilaments (Lynch et al., 2017). In addition, CTPS^H355A^ fails to assemble into the larger, micron-scale structures when expressed in cultured cells (Sun and Liu, 2019). *In vitro*, *Drosophila* CTPS^H355A^ exhibits reduced catalytic activity (Zhou et al., 2019) as does human CTPS1^H355A^ (Lynch et al., 2017), whereas CTPS2^H355A^ exhibits similar activity to wild-type CTPS2 (Lynch and Kollman, 2020). These observations are consistent with the model that H355A blocks assembly in both isoforms but that assembly only enhances catalytic activity of the CTPS1 isoform (Lynch et al., 2017). CTPS has been observed to assemble in diverse eukaryotic organisms and cell lines under conditions of either reduced CTP levels or increased demand for nucleotides (Chen et al., 2011; Noree et al., 2010; Strochlic et al., 2014). Together, these observations suggest that CTPS1 polymerization in eukaryotes may serve as a homeostatic mechanism to increase nucleotide biosynthetic flux during periods when demand outpaces supply.

The *Drosophila* ovary is one of the best characterized metazoan tissues in which assembly of CTPS occurs as part of normal physiology (Aughey et al., 2016; Gou et al., 2014; Wang et al., 2015). Under normal growth conditions, both the germline cells that give rise to the developing egg and the somatic follicle cells that surround the germ cells within the developing egg chambers exhibit CTPS filaments (Liu, 2010; Strochlic et al., 2014). In the germline cells, CTPS filaments assemble transiently during early stages (stages 1–10) of egg development and abruptly disassemble at stage 10, just prior to ‘dumping’, when the cytosolic contents of the 15 ‘nurse’ cells in the germline cyst are transferred to the developing oocyte (Liu, 2010; Strochlic et al., 2014). The developmental period during which CTPS assembles into filaments coincides with a period during which nurse-cell nuclei undergo multiple rounds of endoreplication (Dej and Spradling, 1999) and ribosomal RNA is dramatically upregulated (Mermod et al., 1977), representing a tremendous increase in nucleotide demand. Female hypomorphic mutants of the single gene encoding CTPS in flies are infertile and their ovaries exhibit profound defects in morphology consistent with reduced CTP levels (Strochlic et al., 2014). Furthermore, these phenotypes are rescued by re-expressing a constitutively filament-forming CTPS mutant in the ovarian germ cells (Strochlic et al., 2014), demonstrating a cell-autonomous requirement for CTPS in oogenesis that can be satisfied by polymerized CTPS. These findings demonstrate a biological function of filamentous CTPS but do not test whether assembly per se is essential for this function. The recent identification of the H355A CTPS point mutant (Lynch et al., 2017) and the availability of powerful genetic tools for tissue-specific gene expression in *Drosophila* prompted us to use this system to directly test whether CTPS assembly confers any functional advantage in this native biological context.

## RESULTS

### The H355A mutation disrupts assembly of CTPS *in vivo*

**Fig. 1.**
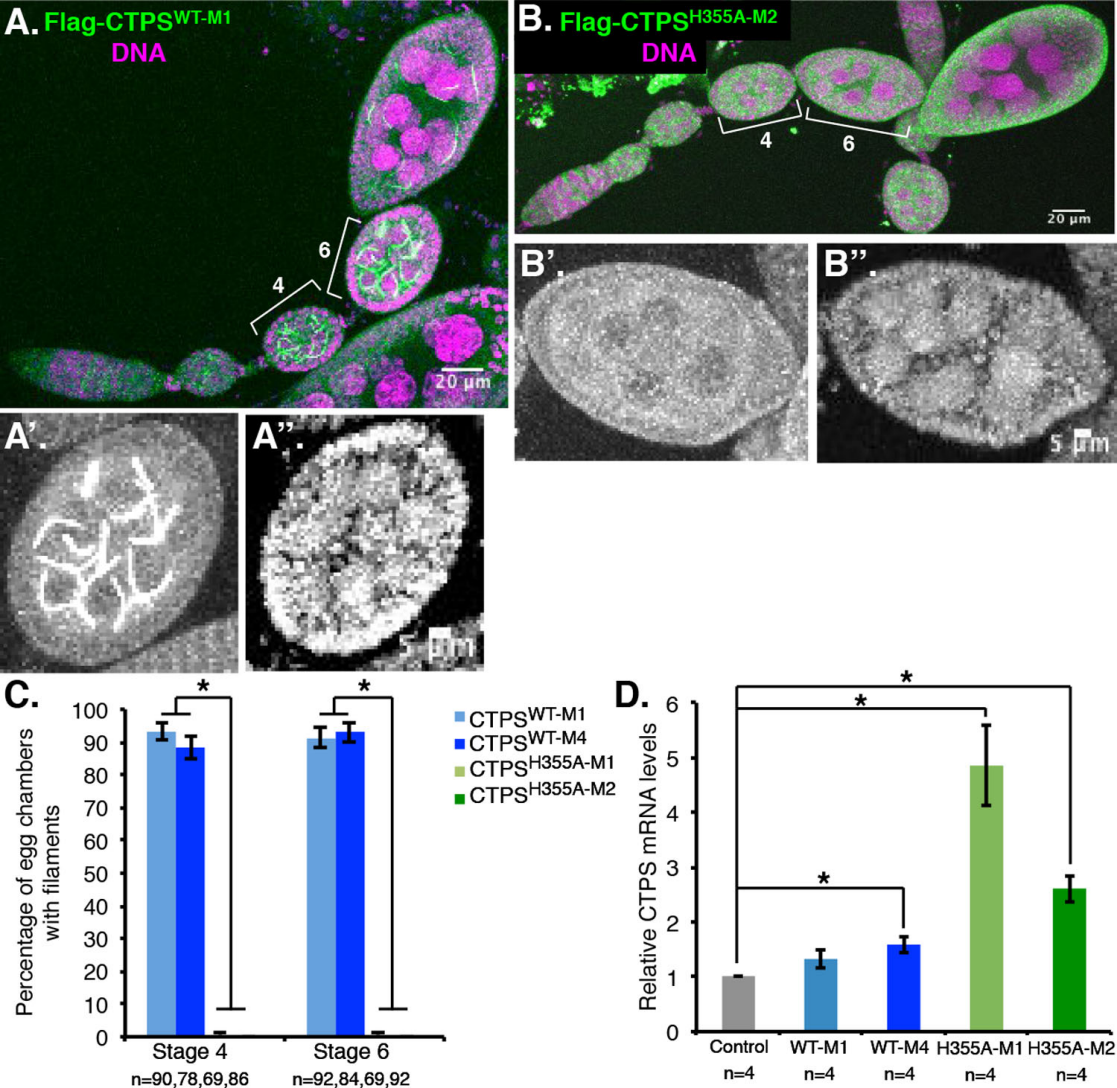
**The *CTPS ^H355A^* mutation prevents CTPS assembly into filaments *in vivo*.** Ovarioles from (A), *Flag-CTPS^WT-M1^/pCOG-Gal4* or (B) *Flag-CTPS^H355A-M2^/pCOG-Gal4* flies were stained with anti-FLAG antibody (green) and Propidium Iodide (magenta) to label DNA. Stage 4 and 6 egg chambers are bracketed for comparison. Lower panels show higher magnification of the stage 6 egg chambers from the A and B images: A′ and B′ are the Flag stain and A″ and B″ are Propidium Iodide. (C) Quantification of the percent of stage 4 and 6 egg chambers with CTPS filaments in two independent *Flag-CTPS^WT^* transgenic lines (M1 and M4) and two *Flag-CTPS^H355A^* lines (M1 and M2). *, *P*<7.5×10^−38^ in comparison with either *Flag-CTPS^WT^* control line by Student's *t*-test; n, number of egg chambers counted for each genotype. Error bars here and in D represent standard error of the mean (s.e.m.). (D) Relative *CTPS* RNA expression (endogenous plus transgene) from ovaries of the indicated genotype was quantified by qRT-PCR. *, *P*-values in comparison to control *pCOG-Gal4* flies are 0.029, 0.013, 0.007 (left to right). *N* denotes the number of independent experiments. Fig. S1 shows similar qRT-PCR data using *FLAG-CTPS* transgene-specific primers.

H355 is conserved between human and *Drosophila* CTPS and is required for higher order assembly of human CTPS tetramers *in vitro* (Lynch et al., 2017). We generated transgenic flies expressing *Drosophila CTPS^WT^* or *CTPS^H355A^* with an N-terminal FLAG tag under UASp control (Rørth, 1998). The pCOG-GAL4 driver was chosen for its expression in germline cells of early stage egg chambers (Rørth, 1998), when CTPS filaments are first evident (Strochlic et al., 2014). Two independent lines for each transgene were characterized to control for transgene integration site differences. FLAG immunostaining revealed that, as expected, CTPS^WT^ was expressed in early egg chambers, where it assembled into filaments morphologically similar to endogenous CTPS filaments (Strochlic et al., 2014) (Fig. 1A). By contrast, CTPS^H355A^ was expressed but did not assemble into filaments (Fig. 1B). Quantification demonstrated robust (>99%) disruption of CTPS assembly by the H355A mutation in stage 4 and 6 egg chambers in both transgenic lines (Fig. 1C). These findings demonstrate the ability of the H355A mutation to disrupt assembly of *Drosophila* CTPS filaments, consistent with a similar study of this mutation in human CTPS1 (Sun and Liu, 2019). Furthermore, it suggests that the ability of CTPS to form protofilaments is an essential step in the assembly of the micron-scale structures observed *in vivo*.

We quantified the expression of *CTPS* by qRT-PCR from whole ovaries to establish the relative levels of expression of transgenes compared to endogenous CTPS as well as to compare levels of expression of the different transgenes. *CTPS* RNA was increased ∼fivefold in *CTPS^H355A-M1^* and ∼threefold in *CTPS^H355A-M2^* lines compared to control ovaries expressing only the pCOG-GAL4 driver (Fig. 1D). *CTPS^WT^* was modestly overexpressed (1.6-fold) in the *CTPS^WT-M4^* line but was not significantly different from control in *CTPS^WT-M1^*. Because template RNA was isolated from whole ovaries, which include additional cell types in addition to germline cells (e.g. follicle cells), these results underestimate the increase in *CTPS* specifically in the germline. We therefore also conducted qRT-PCR using *FLAG-CTPS* specific primers, which showed similar relative expression of the transgenes and confirmed expression of *FLAG-CTPS^WT^* in the *CTPS^WT-M1^* line (Fig. S1). This indicates that the inability of CTPS^H355A^ to assemble was not due to lower expression levels compared to CTPS^WT^ but rather due to the disruption of key intersubunit interactions mediated by this residue in the polymeric state (Lynch et al., 2017).

### CTPS^H355A^ dominantly inhibits assembly of wild-type CTPS

**Fig. 2.**
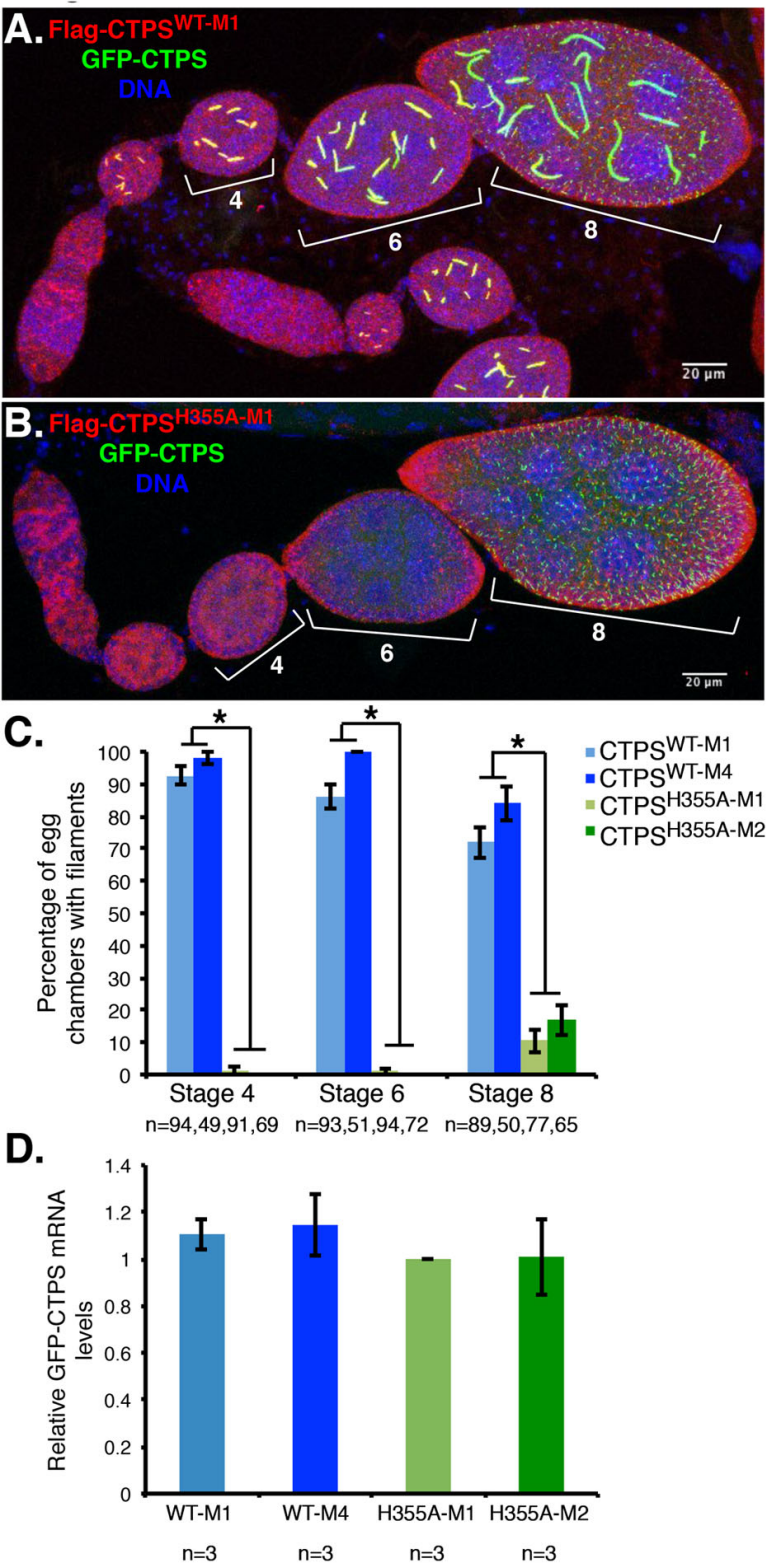
**Transgenic expression of *CTPS^H355A^* dominantly inhibits assembly of endogenous wild-type CTPS.** Ovaries from *GFP-CTPS*-expressing flies also expressing either (A) *Flag-CTPS^WT-M1^* or (B) *Flag-CTPS^H355A-M2^* under *pCOG-Gal4* control were stained with FLAG-specific antibodies (red) and DAPI (blue). Yellow color indicates overlap between red and green channels. Stage 4, 6 and 8 egg chambers are bracketed. (C) Quantification of the percent of stage 4, 6 and 8 egg chambers with CTPS filaments of the indicated genotypes. *, *P*<9.98×10^−14^ in comparison with either *Flag-CTPS^WT^* control line (Student's *t*-test); *n*, number of egg chambers counted for each genotype. Error bars here and below represent s.e.m. (D) Expression of the *GFP-CTPS* reporter was quantified by qRT-PCR from ovaries expressing the indicated transgene. Reporter expression was not significantly different across the four groups (ANOVA *P*=0.7815).

The fundamental unit of CTPS polymerization is the tetramer and CTPS^H355A^ assembles into tetramers structurally indistinguishable from the wild-type protein (Lynch et al., 2017). The possibility that CTPS^H355A^ could co-assemble into tetramers along with the wild-type protein suggested that *CTPS^H355A^* might also exert a dominant negative effect on the assembly of the endogenous wild-type CTPS in transgenic ovaries. To test this, we crossed *CTPS^H355A^* and *CTPS^WT^* transgenes into flies expressing an allele of *CTPS* tagged with GFP (Noree et al., 2010), to allow visualization of endogenous CTPS filaments. While GFP-CTPS filaments in germline cells appeared normal in flies expressing the *CTPS^WT^* transgene (Fig. 2A), *CTPS^H355A-M1^* and *CTPS^H355A-M2^* transgenes both potently disrupted GFP-CTPS assembly (Fig. 2B). Quantification of egg chambers with GFP-labeled filaments in germline cells of stage 4, 6 and 8 egg chambers demonstrated a highly penetrant dominant negative effect of *CTPS^H355A^* expression; almost no stage 4 or 6 egg chambers exhibited GFP-CTPS filaments in *CTPS^H355A-M1^* or *CTPS^H355A-M2^* lines (Fig. 2C). qRT-PCR confirmed that *CTPS^WT^* and *CTPS^H355A^* transgene expression did not differentially alter the expression of the *GFP-CTPS* reporter (Fig. 2D). In stage 8 egg chambers, when pCOG-GAL4-driven transcription sharply decreases (Rørth, 1998), we observed a partial (10–17%) reassembly of GFP-CTPS into filaments (Fig. 2C), likely due to lower CTPS^H355A^ protein levels. We conclude that overexpressed CTPS^H355A^ co-assembles with wild type into tetramers, disrupting the tetramer–tetramer interactions mediated by H355, and thereby prevents assembly of endogenous CTPS protofilaments and larger structures. Thus, both M1 and M2 *CTPS^H355A^* transgenic lines are novel tools to disrupt assembly of endogenously expressed CTPS.

### Disruption of CTPS filaments in early stage egg chamber germline cells impairs fecundity of flies treated with the glutamine antagonist DON

To assess whether expression of *CTPS^H355A^* in all tissues in the fly would have phenotypic consequences, we used *act5c-Gal4* to drive ubiquitous expression of three different UAS-*CTPS^H355A^* alleles. In two independent experiments we crossed female *act5c-Gal4* flies with male flies bearing each of the UAS-*CTPS^H355A^* alleles. In all cases adult progeny bearing both the driver and a *CTPS^H355A^* transgene eclosed in similar numbers to driver or transgene alone controls [for all crosses X^2^ (1, *N*>50)<2.420, *P*-value>0.147]. This indicates that ubiquitous expression of *CTPS^H355A^* does not decrease viability overall. We next focused on effects of *CTPS^H355A^* on fertility.

Flies expressing very low levels of *CTPS* exhibit profoundly reduced female fecundity, decreased germline cell nuclear diameter and loss of germ cell membrane integrity (Strochlic et al., 2014). We therefore examined various phenotypes in pCOG-GAL4-driven *CTPS^H355A^*-expressing flies to specifically assess whether loss of CTPS assembly in the early egg chamber had phenotypic consequences for egg development and fertility. Because flies reared on standard food might have a reduced dependence on *de novo* nucleotide biosynthesis pathways due to the presence of scavengable nucleotides and their metabolites derived from yeast in the food, we also evaluated the role of CTPS assembly in flies reared on food containing the glutamine antimetabolite 6-diazo-5-oxo-L-norleucine (DON). Glutamine is an essential source of nitrogen atoms at two steps of CTP biosynthesis and DON competitively inhibits glutamine-dependent enzymes.

**Fig. 3.**
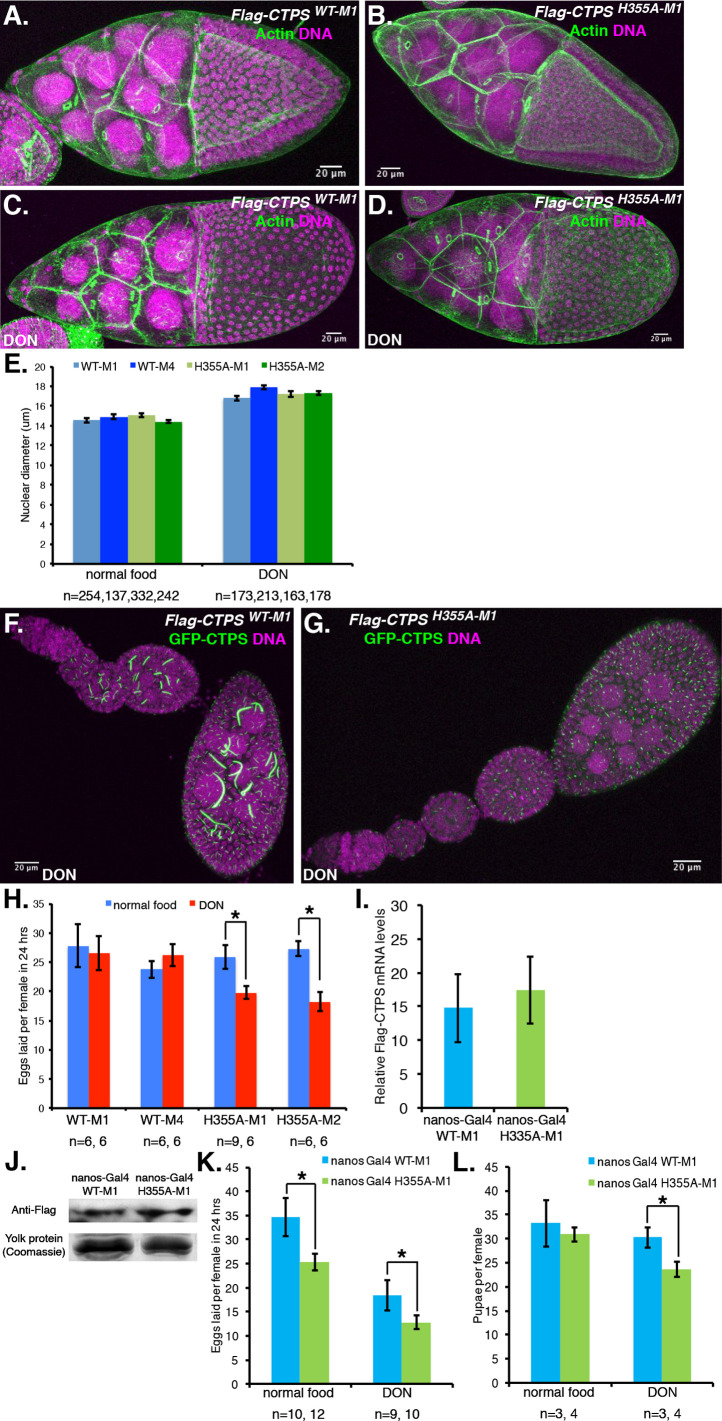
**CTPS polymerization in ovarian germline cells supports egg production.** Representative fluorescent images of stage 10 egg chambers from (A) *Flag-CTPS^WT-M1^* or (B) *Flag-CTPS^H355A-M1^* flies fed standard food stained with Phalloidin-488 (green) to reveal the actin network underlying germline cell plasma membranes. DNA is stained with Propidium Iodide (magenta). (C,D) Similar staining for flies fed food containing the glutamine antimetabolite DON (10 µM). (E) Germline cell nuclear diameter was measured from images of Propidium Iodide-stained nuclei of stage 8 egg chambers of the four genotypes in flies reared on either standard food or DON-containing food. No consistent difference was observed between *Flag-CTPS^WT^* and *Flag-CTPS^H355A^* lines, although DON increased nuclear diameter across all genotypes (Student's *t*-test for all pairwise comparisons *P*<1.11×10^−9^). Number of nuclei counted for each genotype (n) are shown. Here and below, error bars denote s.e.m. (F,G) GFP-CTPS fluorescence (green) and DNA (magenta) in ovarioles of flies expressing the indicated transgenes and fed 10 µM DON-containing food for 4 days. (H) Eggs laid over 24 h per fly expressing the indicated *CTPS* transgene driven by pCOG-GAL4 and fed either standard food or DON-containing food were quantified. *n* indicates the number of times the experiment was replicated. *P*-values (*) left to right were 0.038 and 0.0014. (I) qRT-PCR quantification of *CTPS* transgene expression in ovaries in which the indicated transgene was driven by nosGal4:VP16. was quantified by qRT-PCR. Transgene expression was not significantly different (*t*-test). (J) FLAG western blot of ovaries expressing the indicated FLAG-tagged *CTPS* transgenes as in I. The lower panel shows a corresponding Coomassie-stained gel of the major yolk proteins as a loading control. (K) Egg-laying assay as in H for nosGal4:VP16-driven *Flag-CTPS^WT-M1^* and *Flag-CTPS^H355A-M1^* lines. *P*-values (*) left to right were 0.0358 and 0.0124. (L) Pupae production. Eggs laid over 7 days were allowed to develop in the presence or absence of DON and pupae were counted after 7 days. *P*-values (*) left to right were 0.3223 and 0.0158.

CTP is critical for phospholipid biosynthesis via the Kennedy pathway and *CTPS* hypomorphic mutants with very low CTPS expression show highly penetrant membrane defects in the *Drosophila* ovary that can be visualized by staining the actin network underlying the plasma membrane (Strochlic et al., 2014). We used phalloidin staining to visualize the actin network in stage 10 *CTPS^WT^*- and *CTPS^H355A^*-expressing egg chambers in flies reared on standard food or food containing 10 µM DON. We observed normal morphology in all four transgenic lines, with or without DON treatment (Fig. 3A–D and not shown), suggesting that neither this dose of DON, nor *CTPS^H355A^* expression reduced CTP levels below the threshold required to disrupt membrane architecture. Consistent with this, we also found that nuclear diameter, which is reduced in CTPS-hypomorphic mutants (Strochlic et al., 2014), was not significantly different between *CTPS^WT^*- and *CTPS^H355A^*-expressing stage 8 egg chambers (Fig. 3E, left bars). Unexpectedly, DON treatment increased nuclear diameter in all genotypes (Fig. 3E, right bars), likely because disrupting glutamine metabolism causes broader effects than CTPS inhibition. However, there was no consistent difference between *CTPS^WT^*- and *CTPS^H355A^*-expressing lines. Importantly, DON treatment did not affect the ability of *CTPS^H355A^* to disrupt assembly of GFP-CTPS (compare Fig. 3F and G). Together, these observations suggest that *CTPS^H355A^* disrupts CTPS filament assembly while retaining sufficient CTPS activity to maintain normal subcellular morphology.

Next, we tested whether disruption of CTPS filaments in early stage egg chambers would have consequences for egg production. The number of eggs laid by *CTPS^WT^* and *CTPS^H355A^*-expressing females over a 24 h period were not significantly different (ANOVA *P*=0.6415) between the two genotypes when flies were reared on standard food (Fig. 3H, blue bars), indicating that germline disruption of CTPS assembly between stages 1 and 6 did not affect production of mature eggs under these conditions.

Nucleosides and nucleobases can be obtained dietarily and can also be synthesized *de novo* from precursor metabolites including glutamine. We hypothesized that in flies fed with yeast-containing standard food that both pathways contribute to generating CTP in ovaries and that CTPS assembly in early stage egg chambers is not required to maintain sufficient CTP levels for egg production. Glutamine is required for *de novo* CTP biosynthesis and we imagined that DON-mediated inhibition of this pathway might reduce CTP levels enough to reveal a role for CTPS assembly in supporting oogenesis. Indeed, starvation of adult female *Drosophila* enhances CTPS filament assembly in ovaries (Wu and Liu, 2019). Likewise, feeding flies the glutamine antimetabolites DON or azaserine promotes CTPS filament assembly in many tissues (Chen et al., 2011). We therefore evaluated the relative effect of *CTPS^WT^* and *CTPS^H355A^* expression on egg production by female flies fed food containing 10 µM DON. This dose promoted CTPS assembly in germ cells that persisted even into mature eggs (Fig. S2). The number of eggs laid per fly was significantly decreased by DON in both *CTPS^H355A^* transgenic lines but not in either of the *CTPS^WT^*-expressing lines (Fig. 3H, compare blue and red bars) demonstrating a fecundity advantage of expressing *CTPS^WT^* compared to *CTPS^H355A^*.

To ensure that this phenotypic difference was not due to differences in *CTPS^WT^* and *CTPS^H355A^* expression levels, we screened additional ovary-specific drivers and found that the *nosGal4:VP16* driver gave similar expression of the *CTPS^WT-M1^* and *CTPS^H355A-M1^* transgenes, both at the RNA (Fig. 3I) and protein levels (Fig. 3J). *nosGal4:VP16* is highly expressed throughout all stages of oogenesis (Rørth, 1998), driving ∼2.6-fold higher expression of the *CTPS^H355A-M1^* transgene in the ovary compared to pCOG-Gal4 (not shown). Strikingly, even in the absence of DON, flies expressing *CTPS^H355A-M1^* produced significantly fewer eggs than flies expressing *CTPS^WT-M1^* (Fig. 3K). This defect was even more highly significant in DON-treated flies (*P*=0.0124 versus 0.0358), resulting in a 30% reduction in eggs laid per female. Together, these key findings demonstrate that CTPS assembly supports but is not absolutely essential for egg production under standard laboratory growth conditions and under the stress of inhibited glutamine metabolism.

Finally, we examined the effect of the *CTPS* transgenes on development through the larval stages into pupae in the continuous presence (or absence) of DON. As expected based on the reduced egg laying, *CTPS^H355A-M1^*-expressing females produced fewer pupae than *CTPS^WT-M1^*-expressing controls under DON treatment. (Fig. 3L**)**. In flies fed standard food this comparison did not reach statistical significance, likely because the effects of disrupting CTPS assembly on egg production were less significant in the absence of DON and were further diluted over the subsequent week. Overall, the results demonstrate that disrupting CTPS assembly during early oogenesis results in a pronounced egg-laying defect that can be exacerbated by a glutamine antimetabolite.

## DISCUSSION

This work presents two main novel findings. First, we establish *CTPS^H355A^* as a transgene capable of dominantly inhibiting CTPS polymerization in a living organism without eliminating CTPS catalytic activity, thereby providing a tool for assessing the functional significance of CTPS assembly *in vivo*. Second, using this tool, we tested for the first time the role of CTPS assembly in a biological context in which it occurs as part of normal physiology. We found that CTPS assembly in early stage egg chambers of the *Drosophila* ovary supports egg development and that this phenotype is even more prominent in flies treated with an antimetabolite that suppresses CTP synthesis.

It remains an open question how CTPS assembly enhances egg production. One possibility is that assembly may enhance *Drosophila* CTPS catalytic activity as it does for human CTPS1 (Lynch et al., 2017). This is consistent with the reduced catalytic activity of *Drosophila* CTPS^H355A^ *in vitro* (Zhou et al., 2019). By increasing nucleotide biosynthetic flux, assembly may increase CTP levels as a homeostatic mechanism under glutamine-limited conditions. Consistent with this general hypothesis, glucose starvation in *Saccharomyces cerevisiae* also triggers CTPS filament assembly (Noree et al., 2010). Rather than affecting CTPS catalytic activity, an alternative possibility could be related to negative feedback inhibition. CTPS is competitively inhibited by its product CTP and prior work in *Saccharomyces cerevisiae* suggests that CTP feedback inhibition is an important regulator of CTPS assembly (Noree et al., 2014). If CTPS assembly decreases the affinity of CTPS for CTP, assembly could potentially allow the accumulation of higher CTP levels than would be permitted by unassembled CTPS.

The more significant effect of *CTPS^H355A-M1^* when driven by the *nosGal4:VP16* driver compared to *pCOG-GAL4* is likely due to the broader expression throughout oogenesis (Rørth, 1998), which results in higher levels of ovary transgene expression. The *pCOG-GAL4* driver drives *CTPS^H355A^* transgene expression only up to stage 6 (Rørth, 1998). Beyond this stage, expression of the transgene is reduced, allowing a return of CTPS assembly in later stage egg chambers (Fig. 2C). By contrast, we expect *nosGal4:VP16*-driven *CTPS^H355A-M1^* to suppress CTPS assembly throughout oogenesis. The phenotype caused by *CTPS^H355A^* expression was enhanced by treatment with DON, which suppresses *de novo* CTP synthesis and caused a significant decrease in pupal production, but this was not observed in the absence of DON. This is likely because of a reduced requirement for *de novo* CTP synthesis in flies fed standard food, which contains yeast as a source of nucleotides and their precursors. Consistent with this, we found that ubiquitous expression of *CTPS^H355A^* throughout development did not affect fly viability in flies reared on normal food. Overall, our findings point to a context-dependent defect conferred by a lack of CTPS assembly. This work provides a framework to examine the role of CTPS assembly under other conditions such as nutrient deprivation, which may increase dependence on CTPS assembly.

We examined the role of CTPS assembly specifically in ovarian germline cells, but CTPS filaments are found naturally in numerous *Drosophila* tissues including the brain, testis, trachea, gut and salivary gland (Liu, 2010). Furthermore, nutrient deprivation increases their prevalence in additional tissues (Chen et al., 2011). Tissue-specific expression of the *CTPS^H355A^* allele using available GAL4 drivers can be used to rapidly probe the role of CTPS assembly in these other contexts. Though we found that ubiquitous expression of *CTPS^H355A^* does not decrease fly viability, this does not rule out the possibility of biologically important phenotypes in specific tissues. Furthermore, H355 is conserved in animals as well as both fission and budding yeast, suggesting that CTPS^H355A^ could be useful for perturbing CTPS assembly in diverse genetic model organisms and cell lines.

Assembly into micron-scale structures is an intrinsic property of many metabolic enzymes. A recent study found that 60 of the 440 metabolic enzymes in *S. cerevisiae* can form such structures (Noree et al., 2019). The propensity of metabolic enzymes specifically to form these structures may relate to their enrichment for homo-oligomeric structures, which are poised to evolve such homotypic interactions (Garcia-Seisdedos et al., 2017, 2019). While assemblies may readily evolve, how they are harnessed to regulate protein function may be different for each protein. CTPS is a particularly striking example since the seemingly independent evolution of assembly in bacteria and animals has in the first case stabilized a catalytically inactive conformation of the enzyme (Barry et al., 2014), while in the second case assembly is associated with enhanced activity (Lynch et al., 2017; Strochlic et al., 2014). Thus, the development of tools to specifically disrupt assembly is critical for elucidating biological functions of assembly in distinct proteins. Here we demonstrate generally how transgenic expression of non-assembling mutants can be harnessed to dominantly inhibit assembly, an approach likely to be applicable to other polymerizing enzymes.

## MATERIALS AND METHODS

### Fly stocks

All flies were maintained at 25°C and fed a standard diet made with agar, cornmeal, yeast and molasses, unless otherwise noted. The *CTPS* transgenic fly lines were created by PCR cloning of the LD25005 cDNA (*Drosophila* Genomics Resource Center) using the Gateway cloning system into the pDONR-Zeo vector (Thermo Fisher Scientific) and then into the pPW vector from the *Drosophila* Genomics Resource Center. The *H355A* mutant was created by site-directed mutagenesis using the Pfu Ultra II HS DNA Polymerase (Agilent) and primers 5′-TGAGCCGAGCAAGTACGCCAAGGAGTGGCAGAAG-3′ and 5′-CTTCTGCCACTCCTTGGCGTACTTGCTCGGCTCA-3′. pPW constructs were used to generate transgenic flies (BestGene) and transgene expression was driven in the germline cells of the ovary with pCOG-Gal4 (Strochlic et al., 2014) or nosGal4:VP16 (Bloomington stock #4937). The *CTPS*-*GFP* protein trap line, CA06746, was provided by A. Spradling.

### Immunofluorescence and imaging

Ovaries were dissected in cold phosphate-buffered saline (PBS), fixed with 4% formaldehyde/PBS at room temperature and stained with anti-Flag antibodies (Sigma-Aldrich, MAB3118) overnight at 4°C. Anti-FLAG staining was visualized using goat anti-mouse Cy3 (Jackson ImmunoResearch 715-165-151) for 2 h at room temperature and DNA was counterstained using either Propidium Iodide or DAPI. For actin staining, Phalloidin 488 (Invitrogen A12379) was used. 3D confocal stacks were captured on a Leica confocal SP4 and are presented as maximum intensity Z-projections. Nuclear diameters of the stage 8 germline cells were measured using the Fiji image processing program (Schindelin et al., 2012)*.* Data were analyzed using Microsoft Excel and GraphPad Prism 6.

### Quantitative real-time PCR expression analysis

Each RNA sample was collected from ovaries dissected from 15 females of the indicated genotype. Ovaries were dissected in PBS and RNA was isolated using the Qiagen RNeasy mini kit and stored at −80°C. The RNA was then reverse transcribed using Moloney murine leukemia virus reverse transcriptase (Ambion). TaqMan gene expression assays (Life Technologies) were used to amplify *dCTPS1* variant C (NM_168606.1 F: CTGTGGTCTGGATGTAACCTCG, R: CAAAACGTAAACCTCGCCATG), Flag*/dCTPS1* (F: TACAAGGATGACGATGACAAGAAA, R: CTTGATCGAGGTTACATCCAGAC), *GFP* (F: CCCAGTCCGCCCTGAG, R: ACGAACTCCAGCAGGACCA) cDNA. *RpII140* was used as normalizer (F: CGCACGTGGAAGTTGGTAAT, R: ACAATCAGAGTCCGCGTAACA). The slopes of the standard curves used to convert cycle threshold values to quantities were between −3.2 and −3.7 cycle/log decade.

### Egg and pupae production assays

Ten virgin female flies were reared on standard food or standard food containing 10 µM DON with three *w^1118^* males at 25°C for 7 days. They were then transferred to a grape juice–agar plate (3% agar in 50% grape juice in water) with yeast for 24 h and then eggs laid were counted. In parallel, eggs laid during the 7-day incubation were maintained at 25°C for another 7 days and then pupae were counted.

### Western blotting

Seven–ten ovaries per genotype were manually dissected and lysed in RIPA buffer and extracted proteins were resolved by SDS polyacrylamide gel electrophoresis. Proteins were visualized by Coomassie staining as a loading control or were transferred to nitrocellulose and blotted with 1:500 mouse anti-FLAG antibody (Sigma-Aldrich) to detect the epitope-tagged transgenes.

### Statistics

Unless otherwise indicated, statistical comparisons between cohorts were evaluated using two-tailed Student's *t*-tests with *P*<0.05 used as a threshold for significance.

## Supplementary Material

Supplementary information
