# Supplementary material for: CTP synthase polymerization in germline cells of the developing Drosophila egg supports egg production
Source: Biol Open. 2020 Jul 21;9(7):bio050328. doi: 10.1242/bio.050328 (PMC7390647; doi:10.1242/bio.050328)
Supplement: Supplementary information [file biolopen-9-050328-s1.pdf]

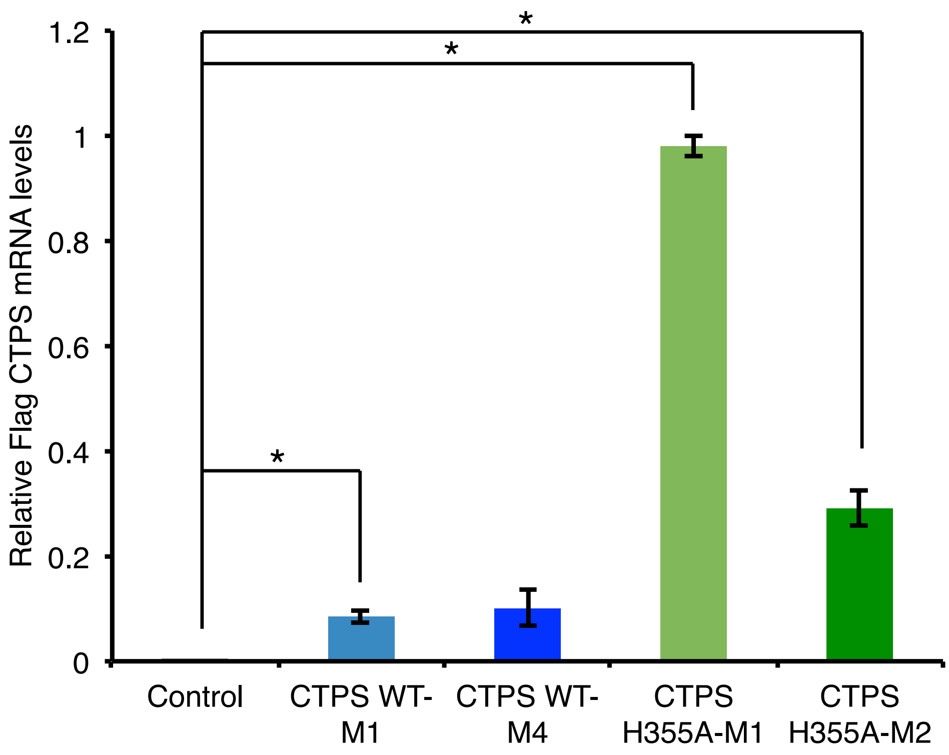

Figure S1: Relative quantification by qRT-PCR of *FLAG-CTPS* transgene expression in ovaries using FLAG-specific primers.
Mean of four independent experiments and s.e.m. are shown. * p values in comparison to control *pCOG-Gal4* flies are .0051, .000017, .0029 (left to right).

Biology Open • Supplementary information

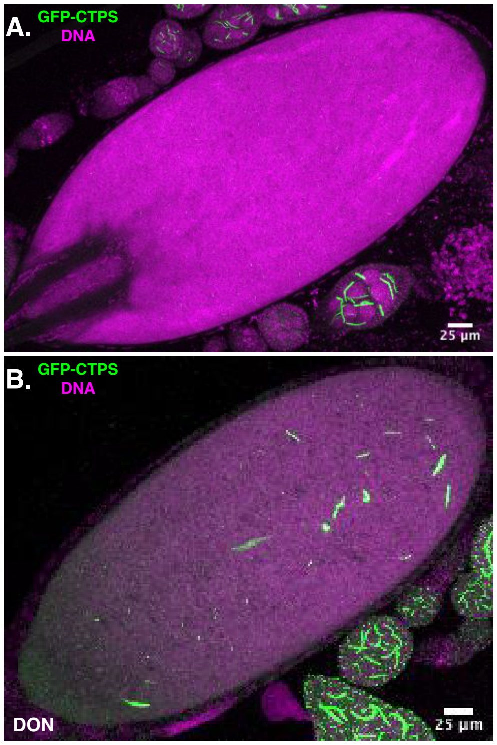

Figure S2: DON treatment promotes CTPS assembly even in late stages of oogenesis.
Ovaries of GFP-CTPS flies either fed normal food (top) or food containing 10 µM DON (bottom) were fixed and stained for DNA with propidium iodide. GFP-CTPS is visualized in green. Mature eggs are shown to illustrate DON-induced assembly of CTPS during late stages of oogenesis.
